# FCGBP Is a Prognostic Biomarker and Associated With Immune Infiltration in Glioma

**DOI:** 10.3389/fonc.2021.769033

**Published:** 2022-01-03

**Authors:** Tengfeng Yan, Daofeng Tian, Junhui Chen, Yinqiu Tan, Yue Cheng, Liguo Ye, Gang Deng, Baohui Liu, Fanen Yuan, Shenqi Zhang, Linzhi Cai, Qianxue Chen

**Affiliations:** ^1^ Department of Neurosurgery, Renmin Hospital of Wuhan University, Wuhan, China; ^2^ Department of Radiology, Wuxi Clinical College of Nantong University, Nantong, China

**Keywords:** FCGBP, glioma, immune infiltration, prognostic biomarker, PD-L1

## Abstract

The Fc Fragment of IgG Binding Protein (FCGBP) has been proven to participate in intestinal tumor immunity. However, the biological role of FCGBP has remained unclear in glioma. The differential expression of FCGBP was explored by Oncomine and GEPIA databases. The effect of FCGBP on prognosis was analyzed *via* Kaplan–Meier plotter and GEPIA. The Tumor Immune Estimation Resource (TIMER) tool was used to determine the correlations of FCGBP expression with tumor immune infiltration. Firstly, FCGBP was highly expressed in glioma and correlated with a worse prognosis. Gene Ontology (GO) and KEGG pathway enrichment analyses revealed that the differentially expressed genes (DEGs) and co-expression genes of FCGBP were mainly involved in the immune response. Furthermore, FCGBP expression was positively associated with multiple immune cells infiltrates as well as the expression levels of multiple immune markers in glioma. FCGBP co-expression networks mostly participated in the regulation of immune response. Finally, immunohistochemistry (IHC) assays were conducted to explore the expression of FCGBP, PD-L1, CCL2 and CD8 in glioma and correlations between them. We found that PDL1 and FCGBP were synchronously upregulated in glioma tissues. These findings revealed a new mechanism by which FCGBP participates in the immune tolerance of glioma, and implied the potential of FCGBP as a therapeutic target or predictive marker for patients.

## Introduction

Glioma is the most common malignant tumor of the brain and central nervous system (CNS) in adults (1). High-grade glioma is significantly aggressive and highly heterogeneous, and their tumor microenvironment contains immune cells, glioma stem cells, and mesenchymal cells (2–4). With the further clarification of molecular markers, in 2021, the World Health Organization (WHO) updated the classification of CNS tumors, which is conducive to the molecular treatment of glioma (5). Therefore, it is particularly important to explore novel effective molecular markers to better guide the diagnosis and treatment of glioma.

The Fc Fragment of IgG Binding Protein (FCGBP) protein has multiple von Willebrand D (VWD) domains that can bind to gel-forming mucins (6, 7). FCGBP is also a cysteine-rich protein, which has been detected in body fluids (8). However, its molecular function is still unclear and may be related to the body’s innate immunity (9, 10). In cancer, FCGBP has been widely found to be differentially low-expressed (11–13). However, the role of FCGBP is still unknown in glioma.

Tumor microenvironment (TME) is an essential element in tumor development, and consists of numerous components, which together determine the final fate of tumor cells (14). Recently studies have shown that tumor-infiltrating immune cells play a leading role in the TME (15). Immunotherapy mainly focuses on the signal axis of immune checkpoint PD-1 and its ligand PD-L1. They are highly expressed in tumors and can bind to PD-1 on the surface of T cells to limit T cell activation and induce depleted state, leading to tumor immunity escape (16).

In the present study, we aimed to identify the expression of FCGBP and its correlation with prognosis, and possible signaling pathway in glioma. In addition, we used the bioinformatics analysis–based immune cell-specific signatures to examin the relationship between FCGBP expression with tumor-infiltrating immune cells and immune-related molecules, and drew further interest regarding whether FCGBP could act as a novel immune marker for immune therapy of glioma patients.

## Materials And Methods

### Human Tissue Samples

We used paraffin-embedded glioma tissue microarrays (108 glioma samples). All specimens were taken from inpatients in the Department of Neurosurgery of Wuhan University People’s Hospital from January 2016 to March 2018. Among these patients, none had received any chemotherapy or radiotherapy before surgery. All patients signed an informed consent form. This study was approved by the Ethics Committee of Wuhan University People’s Hospital School of Medicine.

### Immunohistochemical (IHC) Staining

The paraffin-embedded tissue microarray is heated in an oven (60°C) for 90 minutes. Then, put the slides in different concentrations (100%, 95%, and 75%) of xylene (3 × 5 minutes/time) and ethanol for hydration. After washing 3 times with PBS, 3% H_2_O_2_ was added to the slide and left at room temperature for 10 minutes. Then completely immerse the slide in 95°C antigen retrieval solution for 10 minutes, and then let it cool naturally. Triton-PBS (100X) was used for 5 minutes, and the slides were blocked with 1% BSA for 30 minutes. After that, add the primary antibody and incubate overnight at 4°C. The next day, the slides were washed with PBS (3×10 minutes/time), and then incubated with HRP goat anti-rabbit/mouse IgG for 1 hour. After that, DAB was dropped on the glass slide, and the color reaction was terminated with tap water. Hematoxylin staining was repeated for 1 min, and the color was separated with 1% hydrochloric acid ethanol solution. Finally, cover the glass slide with neutral balsam and Olympus BX40 microscope (Tokyo, Japan) to acquire images.

### Public Database

The mRNA sequencing data and clinicopathological data of all cases in this study were downloaded the Chinese Glioma Genome Atlas (CGGA, http://www.cgga.org.cn/) and the Cancer Genome Atlas (TCGA, https://tcga-data.nci.nih.gov/tcga/) database. The mRNA expression level of FCGBP gene in glioma was explored on the oncomine platform (www.oncomine.org). GeneMANIA (http://genemania.org/) is a gene network visualization website that was used amount of functional correlation genes to identify a single gene, and predicted gene function and related pathways. A p-value less than 0.05 indicates statistical significance. Identify the genomic changes of FCGBP in TCGA-GBM and TCGA-LGG on the cBioPortal platform (http://www.cbioportal.org/). Tumor Immunity Estimation Resource (TIMER, https://cistrome.shinyapps.io/timer/) is a TCGA-based website for systematic analysis of the correlation between genes and different immune cell subtypes in different types of cancer and immune molecules. LinkedOmics (http://www.linkedomics.org/login.php) was used to identify differentially expressed genes related to FCGBP (n = 544) in TCGA-GBMLGG. Differentially expressed genes were analyzed using Pearson correlation coefficient. And enriched it in Gene Ontology (GO) and KEGG pathway. TISIDB is a website for gene- and tumor-immune interaction (http://cis.hku.hk/TISIDB/index.php). The detailed clinical characteristics of the included observations are shown in **Supplementary Tables 1, 2**.

### Gene Set Enrichment Analysis (GSEA)

GSEA (http://software.broadinstitute.org/gsea/index.jsp) was used to analyze potential genes related to FCGBP. According to the true pivot genes (top 50% and bottom 50%), they were divided into high and low groups. Then GSEA was used for bio-process enrichment, and used Normalized Enrichment Score (NES) to estimate GSEA enrichment. The significance of enrichment was evaluated at the FDR <0.25 level, p-value <0.05, and FDR <0.25 level.

### Statistical Analysis

Spearman correlation analysis was used to test the correlation. According to the 50% cut-off point of gene expression, patients were divided into high group and low group. The difference in survival rate between groups was evaluated by Kaplan-Meier survival analysis and log-rank significance test.

## Results

### The Expression Profile of FCGBP in Cancers

**Figure 1:**
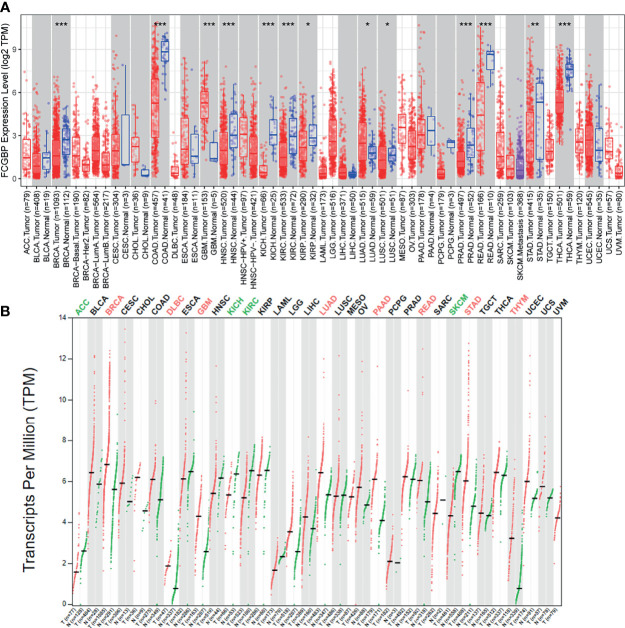
The expression FCGBP in different cancer tissues **(A)** The mRNA expression levels of FCGBP in different tumor tissues and normal tissues analyzed by TIMER (*P <.05, **P <.01, ***P <.001). **(B)** Transcription level of FCGBP was determined in different tumor tissues and normal tissues by GEPIA (*P <.05, **P <.01, ***P <.001).

Firstly, we used TIMER data to evaluate the mRNA expression level of FCGBP in different tumors and normal tissues. TIMER data analysis revealed that FCGBP was significantly lower expressed in most tumor tissues compared with normal tissues. However, FCGBP showed a significantly high expression in glioblastoma (GBM) compared with normal tissues (**Figure 1A**). Through the GEPIA database, we evaluated the transcription level of FCGBP in different tumor tissues and normal tissues (**Figure 1B**). The results showed that the transcription level of FCGBP in GBM and low-grade glioma (LGG) tissues was significantly higher than normal tissues.

### FCGBP Is Up-Regulated in Glioma

**Figure 2:**
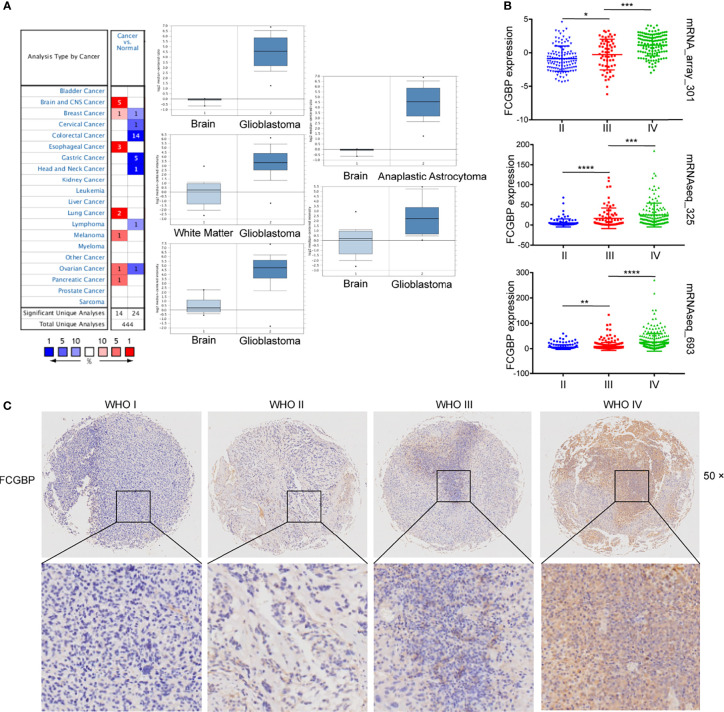
FCGBP is up-regulated in glioma. **(A)** The mRNA expression level of FCGBP in different tumor tissues and normal tissues analyzed by Oncomine (*P <.05, **P <.01, ***P <.001, ****P <0.0001). **(B)** The mRNA expression level of FCGBP was determined in glioma tissues by CGGA database (*P <.05, **P <.01, ***P <.001). **(C)** Typical images of IHC staining of FCGBP in glioma tissues.

Oncomine online database was utilized to analyze the mRNA expression level of FCGBP in tumor and normal tissues. A total of 38 data sets were obtained, and the expression of FCGBP was up-regulated in 14 data sets. Especially in the central nervous system (CNS), the expression of FCGBP in five glioma datasets were all up-regulated (**Figure 2A**). Three independent cohorts including 1319 cases were validated in further study [693 cases in Validation cohort 1 (CGGA-693); 301 cases in Validation cohort 2 (CGGA-301); and 325 cases in Validation cohort 3 (CGGA-325)]. Then, all CGGA database analysis found that FCGBP was also significantly up-regulated in glioma, and positively correlated with the grade of glioma (**Figure 2B**). To verify the upregulation of FCGBP in gliomas, we performed IHC analysis using glioma samples. FCGBP is not detectable in normal brain tissue, but it is expressed in different grades of gliomas, especially high-grade gliomas (**Figure 2C**).

### Prognostic Potential of FCGBP in Glioma

**Figure 3:**
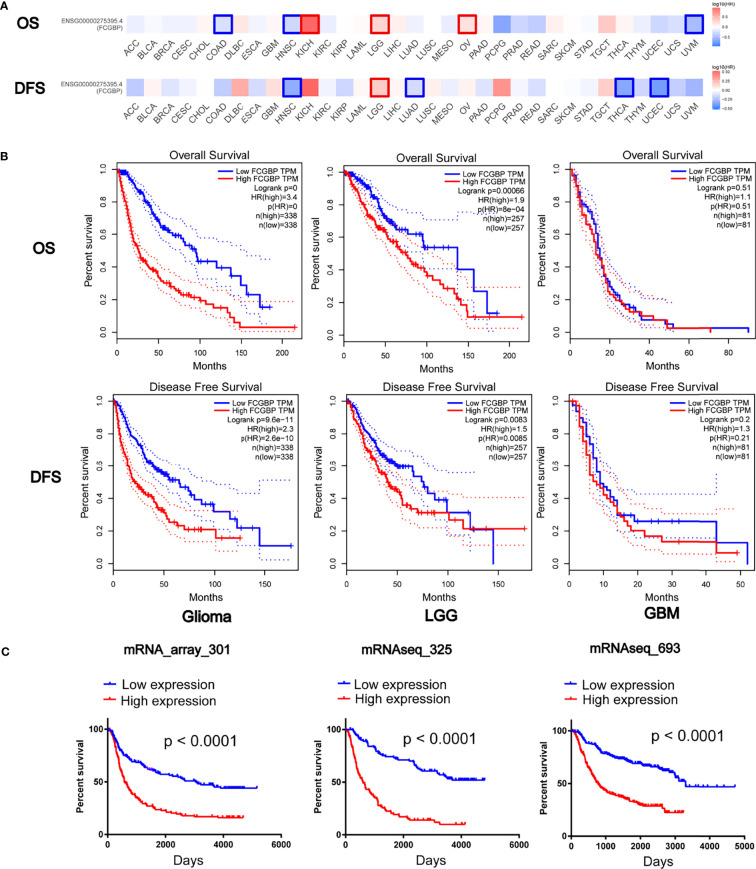
Prognostic potential of FCGBP in glioma. **(A)** The OS and DFS Map of FCGBP in different cancers by GEPIA. **(B)** Kaplan‐Meier survival analysis of all glioma, LGG and GBM in TCGA data sets. **(C)** Kaplan‐Meier survival analysis of all glioma in CGGA data sets.

Further univariate and multivariate Cox regression analysis showed that FCGBP can be significantly negatively correlated with the prognosis of glioma patients in TCGA and CGGA (**Table 1** and **2**). Using an online software GEPIA, which can be used to analyze the survival data from TCGA data sets. Survival analysis showed that the expression of FCGBP was significantly correlated with the poor overall survival (OS) and disease-free survival (DFS) of LGG patients, while high FCGBP expression showed no associations with OS or DFS in GBM patients (**Figure 3A**). Meanwhile, we next scanned the TCGA database, and found that overexpression of FCGBP predicted poor DFS and OS in LGG patients, while high FCGBP expression also showed no associations with OS or DFS in GBM patients (**Figure 3B**). Survival curve analysis was verified *via* three independent CGGA cohorts, and revealed that high expression of FCGBP had a significant poor prognosis in glioma (**Figure 3C**).

**Table 1:** Cox regression analysis of the clinical variables, and overall survival in TCGA cohorts.

Variables	Univariate analysis		Multivariate analysis	
	HR (95% CI for HR)	*P* value	HR (95% CI for HR)	*P* value
FCGBP PRS_type	1.2 (1.2-1.3) 1.2 (0.68-2.2)	0.00* 0.49	0.98 (0.92-1.04) 0.89 (0.44-1.8)	0.47 0.74
Histology	2.5 (2.2-2.9)	0.00*	1.02 (0.81-1.29)	0.87
Grade	4.7 (3.9-5.9)	0.00*	1.9 (1.3-2.76)	0.00*
Gender	1.2 (0.93-1.6)	0.16	1.12 (0.81-1.54)	0.50
Age	1.1 (1.1-1.1)	0.00*	1.04 (1.03-1.06)	0.00*
IDH_mutation	10 (7.5-14)	0.00*	2.78 (1.61-4.79)	0.00*
1p19q_codeletion	4.6 (2.9-7.3)	0.00*	1.87 (1.02-3.43)	0.04*
MGMTp_methylation	3.3 (2.4-4.4)	0.00*	1.32 (0.91-1.9)	0.14

HR, hazard ratio; CI, confidence interval; TCGA, The Cancer Genome Atlas; IDH, isocitrate dehydrogenase; MGMTp, O6-methylguanine-DNA methyltransferase promoter. “*” and Bold values indicates P-value <0.05.

**Table 2:** Cox regression analysis of the clinical variables, and overall survival in CGGA cohorts.

Variables	Univariate analysis		Multivariate analysis	
	HR (95% CI for HR)	*P* value	HR (95% CI for HR)	*P* value
FCGBP PRS_type	1 (1-1) 1 (0.9-1.2)	0.00* 0.56	1.01 (1.01-1.02) 0.78 (0.43-1.41)	0.00* 0.41
Histology	1 (1-1.1)	0.04*	1.11 (0.95-1.28)	0.18
Grade	1.5 (1.3-1.7)	0.00*	1.32 (1.04-1.67)	0.02*
Gender	0.99 (0.83-1.2)	0.95	1.04 (0.84-1.3)	0.69
Age	1 (1-1)	0.01*	1 (0.99-1.01)	0.64
OS	1 (1-1)	0.03 *	1 (1-1)	0.00*
Radio	1.1 (0.87-1.4)	0.41	1.09 (0.82-1.45)	0.54
Chemo	1.3 (1-1.6)	0.03*	0.92 (0.7-1.2)	0.53
IDH_mutation	1.8 (1.5-2.2)	0.00*	1.46 (1.11-1.93)	0.01*
1p19q_codeletion	3.8 (2.5-5.7)	0.00*	4.68 (2.45-8.91)	0.00*
MGMTp_methylation	0.91 (0.75-1.1)	0.36	0.87 (0.7-1.08)	0.22

HR, hazard ratio; CI, confidence interval; CGGA, Chinese Glioma Genome Atlas; IDH, isocitrate dehydrogenase; MGMTp, O6-methylguanine-DNA methyltransferase promoter. “*” and Bold values indicates P-value < 0.05.

### Analysis of Genetic Changes in Glioma, and Interacting Gene Network of FCGBP

**Figure 4:**
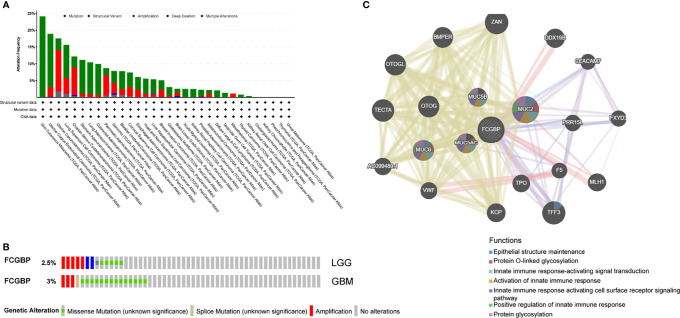
Analysis of genetic changes in glioma, and interacting gene network of FCGBP. **(A)** Genetic alterations of the FCGBP gene were analyzed by cBioPortal. **(B)** The mutation frequency of FCGBP in LGG and GBM. **(C)** FCGBP’s interactive network was generated through the GeneMANIA database.

The cBioPortal online tool was used to analyze the genetic changes of FCGBP in TCGA PanCan Atlas dataset, and we found that FCGBP has a higher mutation frequency in tumors (**Figure 4A**). In LGG and GBM, the results showed a mutation frequency of 2.5% and 3% (**Figure 4B**). The gene-gene interaction network of FCGBP was constructed based on the GeneMANIA database. The functional analysis revealed that FCGBP may related to tumor immune response (**Figure 4C**).

### FCGBP Co-Expression Network in Glioma

**Figure 5:**
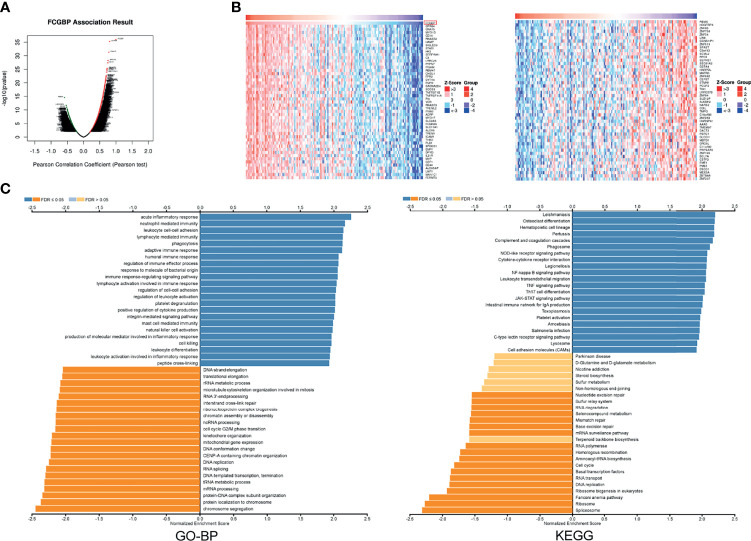
FCGBP co-expression network in glioma **(A)** The volcano map showed FCGBP-related genes in glioma. **(B)** The heatmap showed FCGBP-related genes in glioma. **(C)** Based on the LinkedOmics database, GO annotations and KEGG pathways of genes co-expressed with FCGBP in glioma were significantly enriched.

In order to study the potential function of FCGBP in glioma, we used the LinkedOmics database to explore related molecules of FCGBP. The volcano map shows that red show a significant positive correlation with FCGBP, while green show a significant negative correlation (**Figure 5A**). And select the top 50% genes with positive or negative correlation and display them in the heatmap (**Figure 5B**). GO and KEGG pathway analysis of gene set enrichment analysis (GSEA) was showed that FCGBP co-expressed genes are mainly involved in immune response, etc. (**Figure 5C**). Furthermore, we validated the differentially expressed genes of FCGBP in the CGGA database, and also found that the positively related genes of FCGBP were significantly related to the immune response pathway (**Figure S1**).

### The Relationship Between the Expression of FCGBP and the Level of Immune Infiltration in Glioma

**Figure 6:**
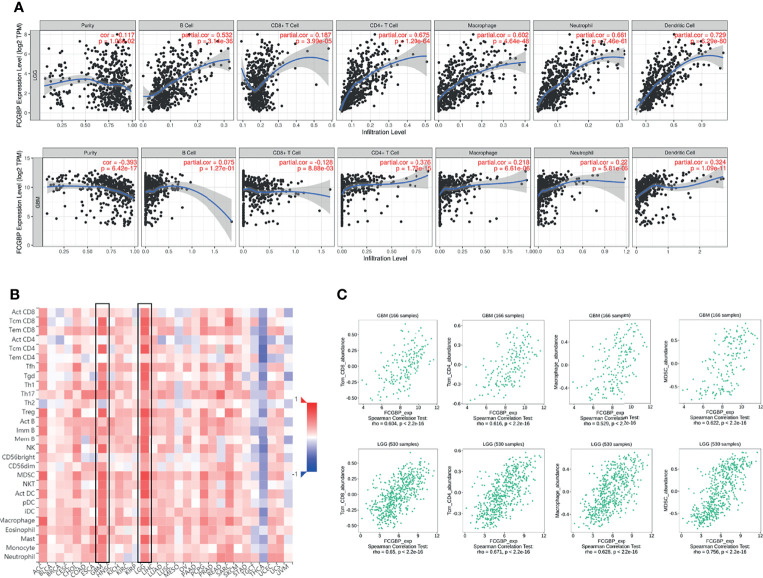
The relationship between the expression of FCGBP and the level of immune infiltration in glioma. **(A)** The expression of FCGBP is correlated with the level of tumor immune infiltration of B cells, CD8 + T cells, CD4 + T cells, Neutrophil and Dendritic cells in glioma. **(B)** Correlations between FCGBP expression and immune infiltration levels in TISIDB. **(C)** The relationship between the expression of FCGBP and CD8 + T cells, effector memory CD4 T cells, Myeloid-derived suppressor cells, macrophages in glioma.

Next, the immune cell infiltration was analyzed using the TIMER website, and we found that the expression of FCGBP was positively correlated with the infiltration of B cells, CD4 T cells, Neutrophil and Dendritic cells (**Figure 6A**). Relationship of FCGBP expression with immune infiltration in tumors was assessed using correlation analysis and TISIDB databases. Furthermore, we found that positive correlation between FCGBP expression and effector memory CD8 ^+^ T cells, effector memory CD4 ^+^ T cells, Myeloid-derived suppressor cells, macrophages (**Figures 6B, C**).

### Correlation Between FCGBP mRNA Expression and Immune-Related Molecules

**Figure 7:**
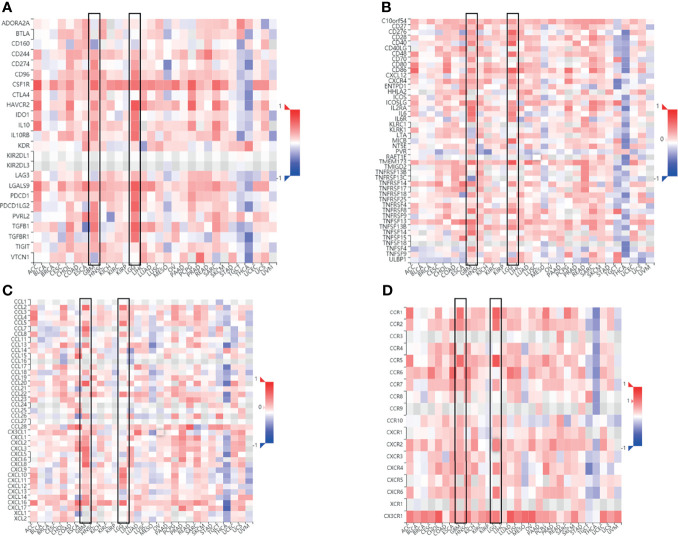
Correlation between FCGBP expression and immune markers. Correlation between FCGBP mRNA expression and immune-related molecules. **(A)** Correlation between FCGBP and glioma immunosuppressive agents. **(B)** The correlation between FCGBP and glioma immunostimulants. **(C)** Correlation between FCGBP and chemokines in gliomas. **(D)** The correlation between FCGBP and glioma receptors.

The Spearman’s correlations between FCGBP expression and immunomodulators, and chemokines were analyzed using the TISIDB database. The results showed the correlation between FCGBP expression level and immunosuppressive agents. The top two immunosuppressive agents include HAVCR2 and TGFB1 (**Figure 7A**). Meanwhile, the correlation between the expression of FCGBP and immunostimulators was analyzed. The top three immunostimulators include CD86, CD40 and TMEM173 (**Figure 7B**). In addition, we also analyzed the correlation between the expression of FCGBP with chemokines and receptors. The top four significantly positively correlated chemokines include CCL2, CCL5, CXCL10, and CXCL16 (**Figure 7C**). For the correlation between FCGBP expression and receptors, the top five receptors include CCR5, CCR1, CXCR6, and CX3CR1 (**Figure 7D**). Therefore, FCGBP could regulate immune molecules.

### Tissue Microarrays (TMAs) and Immunohistochemistry (IHC)

**Figure 8:**
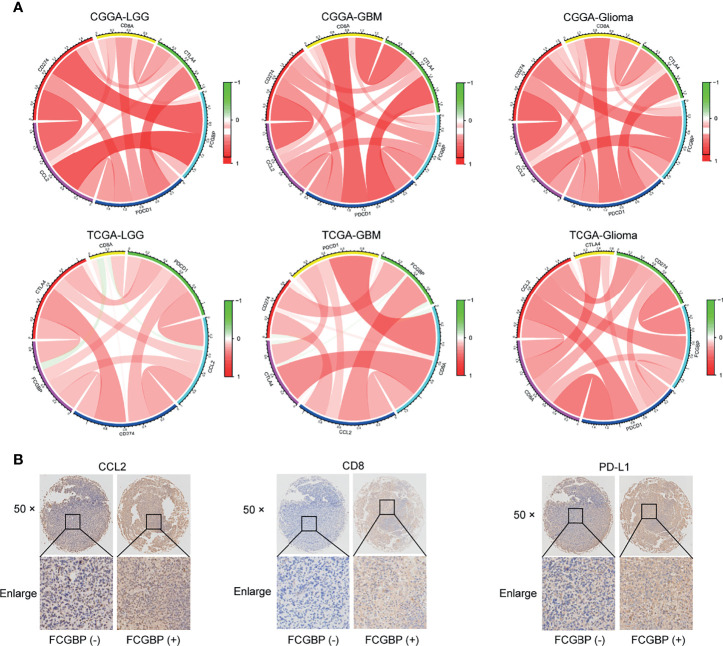
Tissue Microarrays (TMAs) and Immunohistochemistry (IHC). **(A)** The chord diagram illustrating the correlation of the expression of FCGBP with CD8, CCL2 and immune checkpoints molecules (CTLA4, PD1, PDL1). **(B)** IHC staining of CCL2, CD8 and PDL1 in glioma tissues.

Tissue microarrays were constructed as previously described (17). Next, we analyzed the correlation between FCGBP with immune infiltrating molecules and immune checkpoint genes. In addition, the same glioma specimen section was used for CCL2, CD8 and PDL1 IHC staining. The results showed that FCGBP was positively correlated with the expression of CCL2, CD8 and PDL1 (**Figure 8**). These indicate that FCGBP may have a synergistic effect with immune infiltrating molecules and immune checkpoint members.

## Discussion

As an important part of the tumor microenvironment, immune cells have been shown to regulate tumor progression (18). Immunosuppressants began to be gradually used in clinical patients. For example, PD-1 and CTLA-4 antibodies have achieved sustained efficacy in some patients (19). Although new therapies such as immunotherapy have been used, it is still difficult to obtain a good prognosis for GBM patients (20). Tumor heterogeneity may be the main factor limiting GBM patients’ access to effective treatment (21).

FCGBP was previously considered to be one of the components of mucus secreted by goblet cells in the intestine. FCGBP is an important part of the intestinal mucosal immune defense and participates in anti-inflammatory and cell protection (22, 23). Significantly low expression of FCGBP has been observed in intestinal inflammation and tumors, such as ulcerative colitis (24), colorectal adenoma (25), and colorectal cancer (26–28). In addition, FCGBP is also down-regulated in multiple tumors (12, 13, 29, 30), and this imbalance of expression due to different functions that may be involved in different tumors. Through the TCGA and CGGA databases, we first found that FCGBP was highly expressed in glioma tissues and had a significant positive correlation with the grade of glioma. Previous studies have found that ovarian cancer with high FCGBP expression had a worse prognosis, which was consistent with our results (31).

Previous studies have shown that FCGBP may be involved in the immune protection of the intestinal tract (32). Meanwhile, we found that high expression of FCGBP may be a potential poor prognostic factor in this study. Next, KEGG and GO enrichment analysis both show that FCGBP expression is related to immune and inflammation-related biological processes. In the immune microenvironment, tumor-infiltrating immune cells and cancer cells conduct extensive and dynamic crosstalk, which has revealed that certain molecules can participate in this dialogue (33). In this study, we further found that high FCGBP expression is associated with high abundance of immune infiltration, including B cells, CD4+ T cells, neutrophils, macrophages and DCs in LGG. These results indicate at least that FCGBP protein may increase the recruitment of immune cells in LGG, which is not significant in GBM.

The effectiveness of immunotherapy mainly depends on the internal immune microenvironment of the tumor (34). In recent years, immune checkpoint inhibitors have become the most advanced immunotherapy in clinical applications. Among them, death protein 1 (PD-1) and T cell programmed death ligand (PD-L1) have received the most extensive research (35, 36). Through the typical images of immunohistochemistry, we revealed that FCGBP was significantly positively correlated with PD-L1, CD8 and CCL2. This indicated that FCGBP may be related to the up-regulation of immune checkpoints. However, there is no molecular study of FCGBP in glioma cells, further demonstration is needed.

The novelty of this study is that we firstly found that FCGBP has a poor prognostic in glioma, and we also explored the possible mechanism of FCGBP in glioma. We confirmed the correlation between FCGBP and immune infiltration of glioma, and proposed that FCGBP may act a novel immunotherapy biomarker. Therefore, our findings will be helpful to further optimize the immunotherapy of glioma.

## Data Availability Statement

The datasets presented in this study can be found in online repositories. The names of the repository/repositories and accession number(s) can be found in the article/**Supplementary Material**.

## Ethics Statement

All patients signed an informed consent form. The studies involving human participants were reviewed and approved by the Institutional Ethics Committee of the Faculty of Medicine at Renmin Hospital of Wuhan University.

## Author Contributions

Conceptualization, TY, DT, and QC. Data curation, TY, JC, and LC. Formal analysis, TY, JC, LC, LY, YC and QC. Funding acquisition, QC. Investigation, BL and QC. Methodology, SZ. Project administration, DT. Software, TY, JC, and YT. Supervision, BL. Visualization, FY. Writing – original draft, GD. Writing – review and editing, TY and LC. All authors contributed to the article and approved the submitted version.

## Funding

This work was supported by grants from the National Natural Science Foundation of China (NO.82072764). Nantong University School-level Research Fund (NO.2019JQ017) and supported by the Fundamental Research Funds for the Central Universities (NO.2042021kf0133).

## Conflict of Interest

The authors declare that the research was conducted in the absence of any commercial or financial relationships that could be construed as a potential conflict of interest.

## Publisher’s Note

All claims expressed in this article are solely those of the authors and do not necessarily represent those of their affiliated organizations, or those of the publisher, the editors and the reviewers. Any product that may be evaluated in this article, or claim that may be made by its manufacturer, is not guaranteed or endorsed by the publisher.
