# Supplementary figures and images for: FCGBP Is a Prognostic Biomarker and Associated With Immune Infiltration in Glioma
Source: Front Oncol. 2022 Jan 3;11:769033. doi: 10.3389/fonc.2021.769033 (PMC8761730; doi:10.3389/fonc.2021.769033)

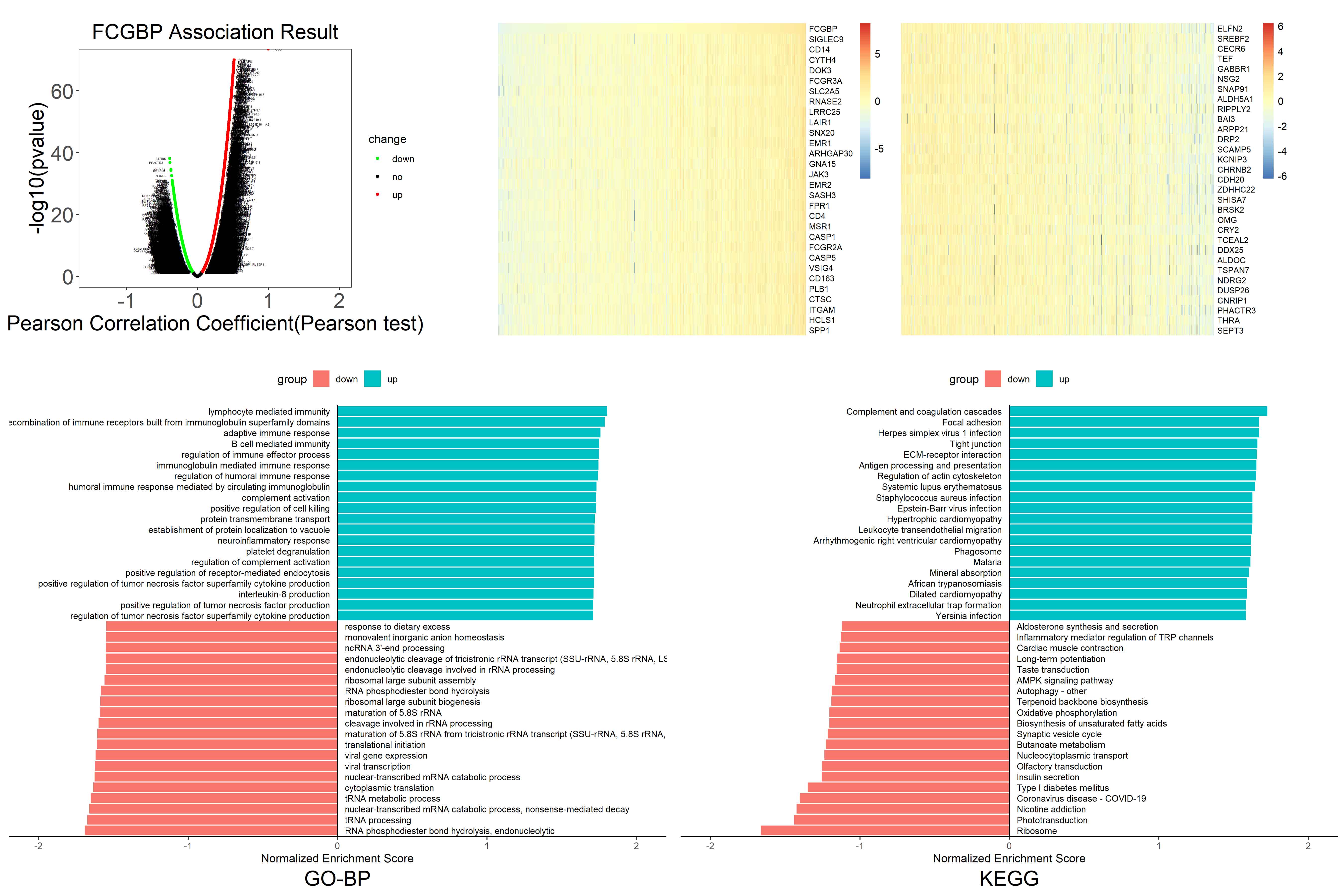

Supplement: Supplementary file 4 [file Image_1.jpeg]
